# Structure Elucidation of Procyanidins Isolated from *Rhododendron formosanum* and Their Anti-Oxidative and Anti-Bacterial Activities

**DOI:** 10.3390/molecules200712787

**Published:** 2015-07-15

**Authors:** Chao-Min Wang, Yuan-Man Hsu, Yun-Lian Jhan, Shang-Jie Tsai, Shi-Xun Lin, Chiu-Hsian Su, Chang-Hung Chou

**Affiliations:** 1 Research Center for Biodiversity, China Medical University, Taichung 40402, Taiwan; E-Mails: wangchaomin@mail.cmu.edu.tw (C.-M.W.); ah_giu@hotmail.com (Y.-L.J.); csungjay@yahoo.com.tw (S.-J.T.); flyalonewithme9147@gmail.com (S.-X.L.); 2 Department of Biological Science and Technology, China Medical University, Taichung 40402, Taiwan; E-Mails: yuanmh@mail.cmu.edu.tw (Y.-M.H.); adaga0806@hotmail.com (C.-H.S.); 3 Department of Life Sciences, National Cheng Kung University, Tainan 701, Taiwan

**Keywords:** procyanidin A1, procyanidin B3, rhodonidin A, procyanidin C4, cinnamtannin D1, anti-bacterial, antioxidant

## Abstract

*Rhododendron formosanum* is an endemic species distributed in the central mountains of Taiwan. In this study, the biological activities of major procyanidins isolated from the leaf extract of *R. formosanum* were investigated. Four compounds, including two procyanidin dimers, procyanidin A1 (**1**) and B3 (**2**), and two procyanidin trimmers, procyanidin C4 (**4**) and cinnamtannin D1 (**5**), were isolated and identified on the basis of spectroscopic data. The structure of a new procyanidin dimer, rhodonidin A (**3**), was elucidated by 2D-NMR, CD spectrum and MS. The procyanidin trimmers and rhodonidin A are reported for the first time in Ericaceae. The biological activities of these procyanidins were evaluated using anti-bacterial and anti-oxidative assays. Only the new compound **3** demonstrated strong anti-bacterial activity against *Staphylococcus aureus* at an MIC value of 4 μg/mL. All compounds showed pronounced antioxidant activities and the activities are enhanced as the amount of OH groups in procyanidins increased. In conclusion, the pleiotropic effects of procyanidins isolated from the leaves of *R. formosanum* can be a source of promising compounds for the development of future pharmacological applications.

## 1. Introduction

Procyanidins are widely distributed throughout the plant kingdom. The evidences linked procyanidins with organoleptic characteristics, plant defense mechanisms, and potential health benefits were reported [1,2,3]. Among plant secondary metabolites, procyanidins are most liable to oxidation and their activity is closely related to plant defense systems against oxidative stress. Moreover, reports of several assays *in vitro* demonstrate potential interactions with biological functions, including antimicrobial [4], anti-proliferation [5], enzyme inhibiting [6], antioxidant, and radical-scavenging properties [1,2]. Typical condensed procyanidins exist as oligomers containing from two to five or six catechin or epicatechin units and as more condensed polymers. However, the structures of procyanidins, particularly larger polymeric procyanidins, are poorly understood.

*Rhododendron formosanum* is an endemic species distributed in the central mountains of Taiwan at elevations from 1500 m to 2500 m. Previously, 18 hydrophobic compounds and two isomeric epoxysitosterols have been isolated and their allelopathic activities were also evaluated [7,8]. Recently, the anti-lung cancer activity of the pentacyclic triterpenoids isolated from *R. formosanum* was reported [9]. Moreover, the hydrophilic compounds responsible for allelopathic phenomenon were also identified by HPLC methods and the major chemical components of the leaves extract of *R. formosanum* were identified as (−)-catechin [10]. Catechin was further transformed into protocatechuic acid in the soil by microbes in the rhizosphere [11].The successful stabilization of *R. formosanum* is due to the synergistic phytotoxic effects of protocatechuic acid and (−)-catechin. Although the major chemicals in the leaves of *R. formosanum* have been investigated prominently, the structures of condensed procyanidins containing catechins or epicatechins units are still unknown.

The aim of this study was to isolate and elucidate the structure of procyanidins from the leaf extract of *R. formosanum*. The biological activities, including antibacterial and antioxidative activities, were also examined.

## 2. Results and Discussion

### 2.1. Identification of Isolated Procyanidins

**Figure 1:**
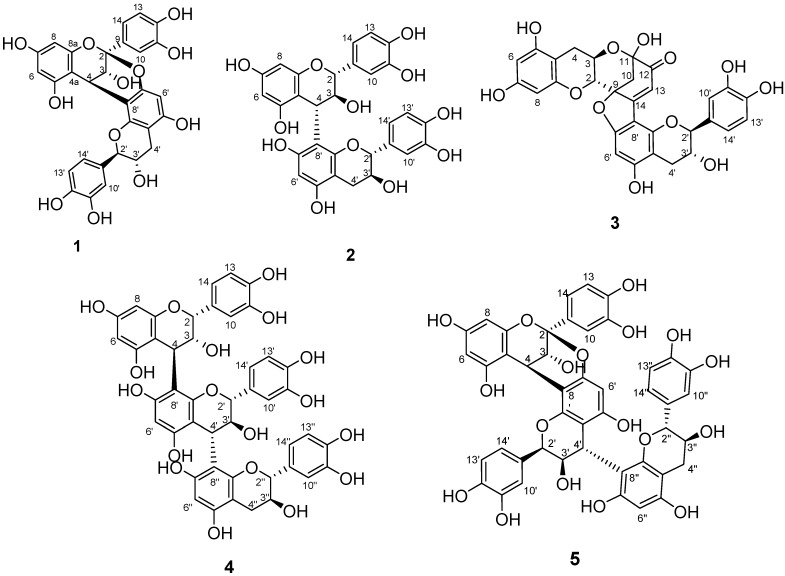
Chemical structures of compounds **1**–**5**.

**Figure 2:**
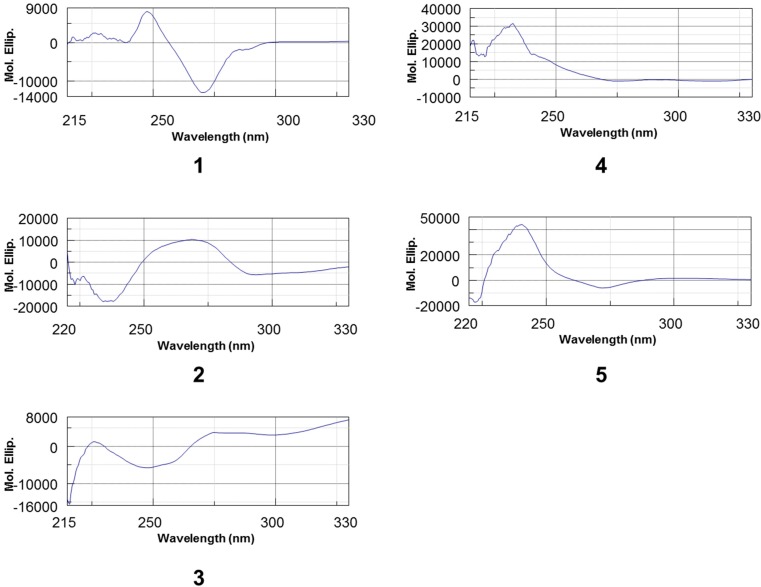
CD spectrum of compounds **1**–**5**.

Chemical structures of compounds **1**–**5** were illustrated in Figure 1. The ESI-MS of compound **1** recorded in negative-ion modes exhibited a deprotonated ion [M − H]^−^ at *m*/*z* 575.1, indicating molecular formulas of C_30_H_24_O_12_. The presence of the isolated AB coupling system at δ_H_ 4.06 (d, *J* = 4.2 Hz, H-3), 4.23 (d, *J* = 3.6 Hz, H-4), the meta-coupled doublets at 5.95, 6.06 (each d, *J* = 2.4 Hz, H-6, H-8), a residual one aromatic proton singlet at δ_H_ 6.08 (s, H-6′), and two AMX systems in the aromatic region (δ_H_ 6.5–7.5) due to rings B and E confirmed the A-type procyanidin. This doubly linked dimeric structure was also supported by the one acetal carbon at δ_C_ 100.3 in its ^13^C-NMR spectrum. A large value of 8–10 Hz for *J*_2,3_ indicates a catechin unit (2,3-*trans*), and a small value of 2 Hz or a broad singlet indicates an epicatechin unit (2,3-*cis*). The signal widths and observable couplings *J*_2,3_ and *J*_3,4_ in **1** indicated the presence of epicatechin and catechin units. In addition, two flavanol units of A-type procyanidins must possess either (2α, 4α) or (2β, 4β) double interflavanyl bonds. The positive Cotton effect at 220–250 nm (Figure 2) of CD spectrum of compound **1** allowed assignment of absolute configuration of C-4 as *R* [12,13], thus deciding the 2β,4β-configuration for compound **1**. Comparison of the ^1^H- and ^13^C-NMR spectroscopic data with the literature established compound **1** as procyanidin A1 (Figure 1), previously isolated from peanut skins [14].

Compound **2** showed a molecular ion with *m*/*z* 577.1 in negative-ion modes, indicating that it was a B-type procyanidin dimer. Two AMX systems in the aromatic region (δ_H_ 5.8–6.9) with large coupling constants in the region of δ_H_ 4.5–3.7 (H-2/H-3/H-4) and the ^13^C-NMR spectrum of two carbon signals at 82.4 and 83.9 corresponding to C2 of C and F rings, two catechin units can be identified. The position of the interflavan bond was determined by HMBC data. CD measurements revealed a negative Cotton effect in the diagnostic wavelength region (220–240 nm), reflecting α-orientation of the 4-flavanyl substituents (Figure 2). Because of rotational and heterocyclic ring conformational heterogeneity in dimeric procyanidins, the proton NMR spectrum of compound **2** exhibited two distinct sets of resonances showing the presence of two rotamers in an approximate 2:1 ratio. Comparison of the ^1^H- and ^13^C-NMR spectroscopic data with the literature established compound **2** as procyanidin B3 (Figure 1) [15].

The HRESI-MS of compound **3** in negative-ion modes showed a deprotonated ion [M − H]^−^ at *m*/*z* 575.1192 (Figure S2), indicating molecular formulas of C_30_H_23_O_12_ (575.1195). The ^1^H-NMR spectrum of compound **3** revealed two AB coupling systems attributable to H-3 atoms (3.98, m; 4.11, m), along with the ^13^C-NMR spectrum of two carbon signals at 79.5 and 83.4 corresponding to C-2 of C and F rings (Table 1). The large value of *J*_2,3_ and *J*_2′,3′_ coupling constant of 8.4 and 7.2 Hz confirmed a 2,3-*trans* configuration of two catechin units. The *meta*-coupled doublets at 5.90, 5.54 (each d, *J* = 3.2 Hz, H-6, H-8), a residual one aromatic proton singlet at δ_H_ 6.13 (s, H-6′), and one AMX systems in the aromatic region (δ_H_ 6.74–6.85) due to rings E confirmed the linkage between two catechin units is from B to D ring. The ^1^H-NMR spectrum of compound **3** presented two singular features concerning the B-ring protons: two duplet (δ_H_ 2.68 and 2.49) with a large coupling constant (*J* = 11.4 Hz) corresponding to the two aliphatic H-10 protons and a singlet (6.43 ppm) corresponding to the H-13 proton involved in a conjugated system. The ^13^C-NMR spectrum of compound **3** exhibited four carbons presenting a chemical shift above 160 ppm corresponding to carbons involved in the conjugated ketone systems of B and D rings. Based on the HMBC correlation (Figures S2–S4, C-11 and C-12 carbons were assigned by H-10 and H-13 protons and their chemical shifts (δ_C_ at 95.3 and 194.1) could be explained by their acetal and ketonic structure.

**Table 1:** ^1^H-NMR (600 MHz) and ^13^C-NMR (150 MHz) spectroscopic data for compound **3** (in CD_3_OD, δ in ppm, *J* in Hz).

Units	Position	^1^H	^13^C
I	2	3.97 d (8.4)	79.5
	3	3.98 m	66.8
	4β	2.94 dd (5.4, 14.4)	27.8
	4α	2.52 dd (9.0, 14.4)	
	4a		100.4
	5		157.6
	6	5.90 (3.2)	97.0
	7		157.9
	8	5.54 (3.3)	95.8
	8a		156.3
	9		89.8
	10	2.68 d (11.4)	45.9
		2.49 d (11.4)	
	11		95.3
	12		194.1
	13	6.43 s	112.8
	14		164.4
II	2′	4.92 d (7.2)	83.4
	3′	4.11 m	67.8
	4′β	2.86 dd (4.8, 16.2)	28.3
	4′α	2.60 dd (7.8, 16.2)	
	4a′		103.9
	5′		166.2
	6′	6.13 s	90.9
	7′		168.0
	8′		105.6
	8a′		155.1
	9′		131.2
	10′	6.85 d (2.4)	114.8
	11′		146.4
	12′		146.5
	13′	6.79 d (8.4)	116.3
	14′	6.74 dd (8.4, 2.4)	119.7

**Figure 3:**
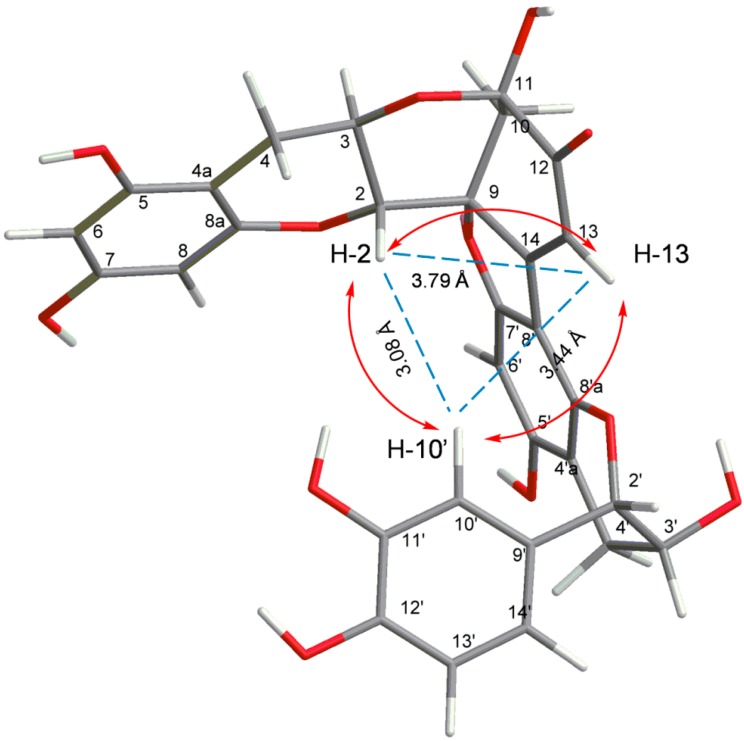
Selected NOESY correlations of compound **3**.

The C-8′ involvement in the interflavan lineage was construed from the HMBC correlations, which permitted us to assign the C-8a′ and the C-5′ carbon atoms. The observation of the HMBC correlation from H-13 to C-8′ also confirmed the linkage between C-14 and C-8′ (Figure S2). In addition, IR spectrum at 1843 and 1714 cm^−1^ also confirmed the ketonoic structure of C-12 (Figure S5). According to the data of ^1^H- and ^13^C-NMR (Table 1) and 2D NMR (HSQC, HMBC), compound **3** is similar to dehydrodicatechin A, a (+)-catechin derivative which had been obtained by enzymatic oxidation [16] and isolated from the roots of *Rosa laevigata* [17] and *Quercus ilex* [18]. However, the NOE correlation between H-2 and H-10′ (Figure S6) indicated the 3D structure of **3** is a compact and not extended form. The three-dimensional structure of compound **3** was obtained using ChemBio3D software and the MM2 force field. In the compact form of (−)-catechin dimer, correlation peaks are observed between H-2 and H-10′, H-10′ and H-13, and H-2 and H-13, for which the interatomic distance measured on the minimized structure are 3.76 Å, 2.98 Å, and 3.32 Å, respectively (Figure 3). In the extended structure of (+)-catechin dimer, the NOE correlation could not be observed because the interatomic distances are all over the detection limited (5 Å). Moreover, circular dichroism is a powerful tool for establishing the absolute configuration of flavonoids and procyanidin. A positive Cotton effect at 280 nm indicated a 2*S* configuration while the negative Cotton effects in the 240 nm region indicated 3*R* absolute configurations, respectively (Figure 2) [19]. The 2*S*, 3*R* configuration was also suggested by the negative optical rotation of **3**. Taking the NOE interactions into consideration, the data of circular dichroism defined the (−)-catechin unit with 2*S* and 3*R* absolute configurations. Thus, the name of compound **3** is given as rhodonidin A (Figure 1).

The ESI-MS of compound **4** in the positive- and negative-ion modes exhibited a sodiated ion [M + H + Na]^+^ at *m*/*z* 890.1 and deprotonated ion [M]^–^ at *m*/*z* 866.2, indicating molecular formulas of C_45_H_38_O_18_, suggested a trimeric B-type procyanidin. The ^1^H NMR spectrum of compound **4** revealed three proton signals (3.98, brs; 4.68, m; 4.14, m) attributable to the H-3 atoms, along with a set of signals due to the H-2 atoms of confirmed the one epicatechin with two catechin units. The ^13^C-NMR spectrum of compound **4** exhibited two C-2 signals at δ_C_ 82.0 and 83.5 due to catechin units and one C-2 signal at δ_C_ 76.1 consistent with an epicatechin unit. The spectroscopic data indicated the lineages between units were connected at position C-4 of unit I/II to C8 of unit II/III, which were confirmed by HMBC correlations between H-4 and C-7′, C-8′, and C-9′ and between H-4′ and C-7′′, C-8′′, and C-9′′, respectively. The CD spectrum of **4** showed a positive Cotton effect at 220–250 nm (Figure 2), demonstrated a β-orientation of 4-flavanyl linkage. According to the data of ^1^H- and ^13^C-NMR and 2D NMR (HSQC, HMBC, COSY, NOESY), compound **4** is defined as procyanidin C4 [20].

The ESI-MS of compound **5** recorded in the positive- and negative-ion modes exhibited a sodiated ion [M + Na]^+^ at *m*/*z* 887.1 and deprotonated ion [M − H]^–^ at *m*/*z* 863.1, indicating molecular formulas of C_45_H_36_O_18_, suggested a triflavonoid moiety (trimeric A-type procyanidin) having only one C–O–C interflavanoid linkage in the structure. All ^1^H- and ^13^C-NMR resonances of compound **5** were assigned by analysis of the 2D NMR (HSQC, HMBC, NOESY) data. In the ^1^H-NMR spectrum, the presence of the AB coupling system at δ_H_ 3.45 and 4.00 (each d, *J* = 3.5 Hz) also indicated an A-type unit in compound **5**. This doubly linked structure was also supported from the one acetal carbon signal at δ_C_ 100.0 in the ^13^C-resonace. The NMR data of compound **5** in GHI moiety appearing at δ_H_ 3.94 (d, *J* = 9 Hz), 3.67 (m), 3.05 (dd, *J* = 16.2, 6.0), and 2.42 (dd, *J* = 16.2, 10.1) and δ_C_ 83.2, 70.0, and 30.6 consistent with the terminal unit were identified as a catechin moiety. The ^1^H and ^13^C spectroscopic data of compound **5** in DEF moiety at δ_H_ 5.51 (brs), 4.06 (d, *J* = 1.8), and δ_C_ 78.6, 72.4 suggested units II is epicatechins. Oligomeric procyanidins are generally linked from C-4 of one flavan unit to C-6 or C-8 of another, and when doubly connected it is often from C-2 of the upper unit to the hydroxyl group of the next unit at C-5 or C-7 position. The lineages between units were confirmed by HMBC correlations between H-4 and C-7′, C-8′, and C-9′ and between H-4′ and C-7′′, C-8′′, and C-9′′, respectively. The CD spectrum of **5** showed a strong positive cotton effect at 220–250 nm, demonstrated a β-orientation of 4-flavanyl linkage (Figure 2) [21]. Comparison of the ^1^H- and ^13^C-NMR spectroscopic data with the literature established compound **5** as cinnamtannin D1 (Figure 1), previously isolated from *Cinnamomum cassia* [22], the leaves of *Machilus philippinensis* [21] and the bark of *Parameria laevigata* [23].

### 2.2. Antibacterial Activity

As shown in Table 2, the antibacterial activities of compounds **1**–**5** were tested against eight bacterial pathogens by minimum inhibitory concentration (MIC) or minimum bactericidal concentration (MBC) methods. Only procyanidins dimer (compound **1**–**3**) displayed antibacterial activities against *S. aureus*. None of the procyanidins trimer showed pronounced antibacterial activities against all tested pathogens. In addition, only compound **1** demonstrated medium antimicrobial activities against *L. monocytogenes* and *B. cereus*. None of the bactericidal activities of isolated compounds against *H. pylori* were observed in this study.

Previous studies revealed a moderate antibacterial activity for certain procyanidins against *Streptococcus pyogenes*, *Bacillus cereus*, *Klebsiella pneumoniae*, and *Proteus vulgaris* at concentrations <100 µg/mL [24]. The determination of MIC against *S. aureus* gave a value of 100 µg/mL for procyanidin B2 [25], a procyanidin dimer with two epicatechin units linked with 4β-8 interfavan bond. In this study, procyanidin A1 (**1**) and B3 (**3**) generated anti gram-positive bacteria activities at MIC values of 64 µg/mL. All these results indicated procyanidin dimers displayed moderate antimicrobial activity against certain pathogens. Structure modification of procyanidins, such as rhodonidin A (**3**), may increase the antibacterial ability against *S. aureus*. In Asia, *S. aureus* is the leading cause of food-born pathogen. Thus, assessing potential antibacterial agent, such as rhodonidin A, and its antibacterial mechanism against *S. aureus* is a hot area of investigation.

**Table 2:** The minimum inhibitory concentration (μg/mL) of antibiotics and natural procyanidins for different bacterial pathogens.

Pathogens	Minimum Inhibitory Concentration (μg/mL)							
	Antibiotics and Procyanidins							
	Ap *	Tet	Met	1	2	3	4	5
*Staphylococcus aureus*	16	8	^+^N.D.	64	64	4	>128	>128
*Enterococcus faecalis*	2	4	N.D.	>128	>128	>128	>128	>128
*Listeria monocytogenes*	1	2	N.D.	64	>128	>128	>128	>128
*Bacillus cereus*	128	4	N.D.	64	>128	>128	>128	>128
*Escherichia coli*	4	0.5	N.D.	>128	>128	>128	>128	>128
*Salmonella enterica*	1	8	N.D.	>128	>128	>128	>128	>128
*Pseudomonas aeruginosa*	512	32	N.D.	>128	>128	>128	>128	>128
Helicobacter pylori **	N.D.	N.D.	2	>256	>256	>256	>256	>256

* Ap: ampicillin; Tet: tetracycline; Met: metronidazole; **1**: procyanidin A1; **2**: procyanidin B3; **3**: rhodonidin A; **4**: procyanidin C4; **5**: cinnamtannin D1; ** *H. pylori* was tested by minimum bactericidal concentration method. ^+^N.D.: not determined.

### 2.3. Antioxidative Activity

**Figure 4:**
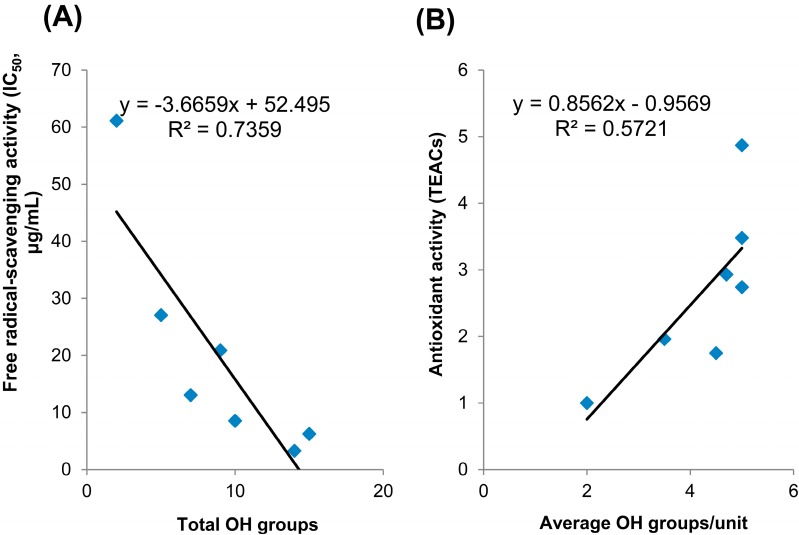
Correlations of total OH groups with free radical-scavenging activity (**A**) and average OH groups/unit with antioxidant activity (**B**).

The antioxidant activities of the isolated procyanidins were measured using the DPPH free radical-scavenging assay and CUPric reducing antioxidant capacity (CUPRAC) method. The results from the DPPH (IC_50_) method for the standard trolox, (−)-catechin and compounds **1**–**5** isolated in this study showed values of 61.12, 27.07, 20.89, 8.55, 13.06, 6.26 and 3.29 μg/mL, respectively (Table 3). Cinnamtannin D1 showed lowest IC_50_ value at 3.29 μg/demonstrating the strongest free radical-scavenging activity in this study. The radical scavenging activity is enhanced as the amount of OH groups in procyanidins increased (Figure 4A). These observations were in line with the results reported previously [24,26]. Ricardo da Silva *et al.* stated that it was not the degree of polymerization, but the number of hydroxyl groups that was important for the radical scavenging activity.

**Table 3:** The antioxidant activities of the procyanidins from leaves of *R. formosanum* using the DPPH free radical-scavenging assay (IC_50_, μM) and CUPric reducing antioxidant capacity (CUPRAC) method (TEACs).

Compounds	Total OH Groups	Average OH/unit	Antioxidant Activity	
			IC_50_/DPPH (μg/mL)	CUPRAC (TEACs)
Trolox	2	2	61.12	1.00
(−)-Catechin	5	5	27.07	2.74
**1**	9	4.5	20.89	1.75
**2**	10	5	8.55	4.87
**3**	7	3.5	13.06	1.96
**4**	15	5	6.26	3.48
**5**	14	4.7	3.29	2.93

In CUPRAC assay, trolox was used as standard chemical for antioxidant activity comparison. B-type procyanidins, such as procyanidin B3 and C4, displayed the highest values of antioxidant activities at 4.87 and 3.48 (TEACs), respectively. In contrast, A-type procyanidins A1 and rhodonidin A showed the lowest value at 1.75 and 1.96 (TEACs), respectively. Our results did not show a pronounced difference in antioxidant activity between total OH groups or the degree of polymerization (data not shown) but a significant increase between the average OH groups/unit with the antioxidant activity (Figure 4B).

## 3. Experimental Section

### 3.1. General Information

Optical rotations were obtained on a Jasco P-2000 digital polarimeter (Jasco, Tokyo, Japan). IR and UV spectra were recorded on Shimadzu IRAffinity 1S spectrometer (Shimadzu Corp., Tokyo, Japan) and Thermo MultiskanGo (Thermo Scientific, Lafayette, CO, USA), respectively. Circular Dichroism was obtained on a Jasco 715 spectrometer (Jasco, Tokyo, Japan). NMR spectra were obtained with a Varian Inova 600 NMR spectrometer (Angilent Tech., Palo Alto, CA, USA). ESI-MS spectra were performed on a Bruker Daltonics Esquire HCT spectrometer (Bruker Daltonics Inc., Billerica, MA, USA). HPLC analysis was carried out on a Hitachi L2130: column, Gemini C6-Phenyl, (5 μm, 10 mm × 250 mm); detector L2420 (Hitachi, Tokyo, Japan). Silica gel 60 (Merck, Darmstadt, Germany), Sephadex LH-20 (GE Healthcare, Uppsala, Sweden), XAD-2 (Sigma-Aldrich, St. Louis, MO, USA), XAD-7 (Sigma-Aldrich, St. Louis, MO, USA), Toyopearl HW-40F (Tosoh Bioscience, Tokyo, Japan), and RP-18 gel (LiChroprep, 40–63 µm, Merck) were used for column chromatography. TLC was carried out on silica gel 60 (Merck, Germany) plates, and spots were visualized under UV light (254 or 356 nm) or by spraying with 5% H_2_SO_4_ in 95% EtOH followed by heating.

### 3.2. Plant Material

The leaves of *Rhododendron formosanum* were collected in April and July of 2010 from the study sites in Yuanzui mountain (24°14′6.49′′ N, 120°57′7.29′′ E at 1911 m a.s.l.) in Hopin township of Taichung County, Taiwan.

### 3.3. Isolation and Identification of Procyanidins

**Figure 5:**
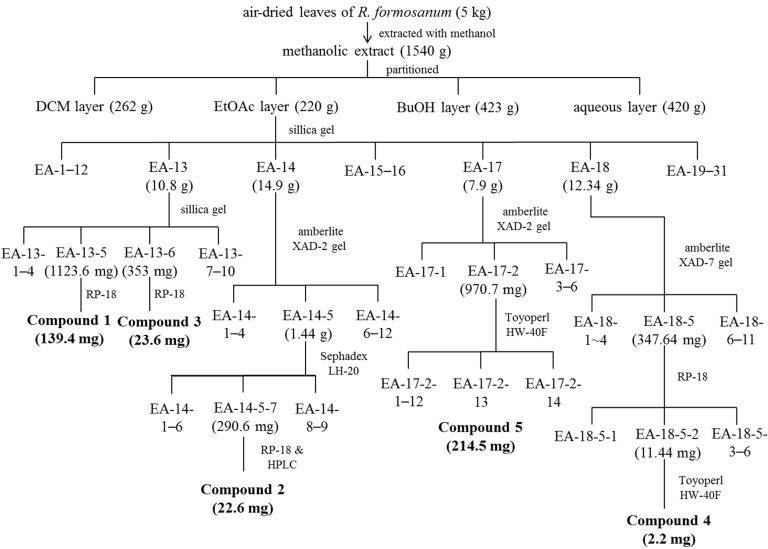
Purification flow chart of procyanidins isolated from *R. formosanum*.

Five kilograms of air-dried leaves of *R. formosanum* was extracted with methanol thrice followed the standard extraction procedures [27]. The methanolic extract was concentrated to obtain 1540 g dry residue and then partitioned by dichloromethane (DCM), ethyl acetate (EtOAc) and *n*-butanol (BuOH) with H_2_O to obtained portion of DCM (262 g), EtOAc (220 g), BuOH (423 g), and aqueous layer (420 g). The EtOAc portion was subjected to a silica gel column in gradient elution of mixture solvent composed of hexane–thyl and acetate–methanol and led to 31 fractions (EA-1–EA-31). Fraction EA-13 (10.8 g) was further subjected to a silica gel in gradient elution of ethyl acetate-methanol and led to 10 subfractions. In gradient elution of MeOH–H_2_O (20%–40%), fraction EA-13-5 (1123.6 mg) was separated via RP-18 chromatography to obtain the compound **1** (139.4 mg). Compound **3** (23.6 mg) was further purified from fraction EA-13-6 (353 mg) by RP-18 chromatography (20% MeOH). In elution of 100% MeOH, fraction 14-5 (1.44 g) was separated by Sephadex LH-20 to give nine subfractions. EA-14-5-7 (290.6 mg) was further subjected to RP-18 (20% MeOH) and compound **2** (22.6 mg) was purified by semi-prepared high-performance liquid chromatography (HPLC: column, Gemini C6-Phenyl, 5 μm, 10 mm × 250 mm; solvent system: acetonitrile-0.2% formic acid with gradient elution; flow rate: 1.5 mL/min; UV detection: 280 nm). In gradient elution of MeOH–H_2_O (60%–100%), fraction EA-17 (7.9 g) was separated via Amberlite XAD-2 gel column to obtain six subfractions. Fraction EA-17-2 (970.7 mg) was further fractionated by Toyopearl HW-40F chromatography in gradient elution of MeOH–H_2_O (40%–100%) to give 14 subfractions. Finally, compound **5** (214.5 mg) was isolated from the subfraction of EA-17-2-13. In addition, fraction EA-18 (12.34 g) was further purified through an Amberlite XAD-7 column (gradient elution of MeOH–H_2_O, 0%–100%), RP-18 column (gradient elution of MeOH–H_2_O, 40%–100%), and a Toyopearl HW-40F column, resulting in compound **4** (2.2 mg). Purification flow chart of procyanidins isolated from *R. formosanum* was illustrated as Figure 5. Purified compounds were subjected to spectroscopic identification by using ^1^H-NMR and ^13^C-NMR (Varian Inova 600) and ESI-MS (Bruker Daltonics Esquire HCT). The isolated compounds were identified by comparison of spectra data with literatures reported previously. The compound purity was further purified by high-performance liquid chromatography (HPLC: column, Gemini C6-Phenyl, 5 μm, 4.6 mm × 250 mm; solvent system: acetonitrile-0.2% formic acid with gradient elution; flow rate: 1 mL/min; UV detection: 280 nm). The purity of isolated compound was over 95%.

*Epicatechin-(4β→8;2β→O→7*)-*catechin* (Procyanidin A1) (**1**). White amorphous powder; ${\left[\alpha \right]}_{D}^{25}$ +18.4° (*c* = 0.1, MeOH); UV (MeOH) λ_max_ (log ε) 280 (2.32) nm; CD (MeOH, *c* = 0.025) nm (ε) 226 (+0.72), 248 (+2.40), 270 (−3.95); IR (KBr) cm^−1^: 3394, 1624, 1521, 1506, 1473, 1452, 1373, 1286, 1178, 1143, 1116, 1066, 1008, 972, 879, 832, 783, 626; ESI-MS *m*/*z* 575.1 [M − H]^–^ (Calcd for C_3__0_H_23_O_12_: 575.1). ^1^H-NMR (CD_3_OD, 600 MHz) δ_H_ 4.06 (1H, d, *J* = 4.2 Hz, H-3), 4.23 (1H, d, *J* = 3.6 Hz, H-4), 5.95 (1H, d, *J* = 2.4 Hz, H-6), 6.06 (1H, d, *J* = 2.4 Hz, H-8), 7.12 (1H, d, *J* = 1.8 Hz H-10), 6.81 (1H, d, *J* = 8.4 Hz, H-13), 7.01 (1H, dd, *J* = 8.4, 2.4 Hz, H-14), 4.72 (1H, d, *J* = 7.8 Hz H-2′), 4.14 (1H, m, H-3′), 2.57 (1H, dd, *J* = 16.2, 8.4 Hz, H-4′α), 2.94 (1H, dd, *J* = 16.2, 5.4 Hz, H-4′β), 6.08 (1H, s, H-6′), 6.91 (1H, s, H-10ʹ), 6.81 (1H, s, H-13′), 6.81 (1H, d, *J* = 8.4 Hz, H-14′); ^13^C-NMR (CD_3_OD, 150 MHz) δ_C_ 100.3 (C-2), 67.8 (C-3), 29.2 (C-4), 104.0 (C-4a), 156.8 (C-5), 98.1 (C-6), 158.1 (C-7), 96.5 (C-8), 154.2 (8a), 132.3 (C-9), 115.6 (C-10), 146.8 (C-11), 145.6 (C-12), 116.3 (C-13), 119.8 (C-14), 84.5 (C-2′), 68.1 (C-3′), 29.0 (C-4′), 103.1 (C-4′a), 156.1 ( C-5′), 96.5 (C-6′), 152.2 (C-7′), 106.8 (C-8′), 151.4 (C-8′a), 130.5 (C-9′), 115.7 (C-10′), 146.8 (C-11′), 146.3 (C-12′), 115.7 (C-13′), 120.7 (C-14′).

*Epicatechin-(4α→8)-catechin* (Procyanidin B3) (**2**). White amorphous powder; ${\left[\alpha \right]}_{D}^{25}$ +14° (*c* = 0.1, MeOH); UV (MeOH) λ_max_ (log ε) 208 (2.74), 236 (2.59) nm; CD (MeOH, *c* = 0.025) nm (ε) 214 (+2.73), 234 (−0.74), 270 (0.43); IR (KBr) cm^−1^: 3404, 1614, 1558, 1508, 1489, 1456, 1373, 1338, 1284, 1145, 1107, 1064, 817, 516, 424; ESI-MS: [M − H]^–^, 577.1 *m*/*z*, (calcd for C_30_H_25_O_12_: 577.1); ^1^H-NMR (CD_3_OD, 600 MHz, 2:1 mixture of rotational isomer), major isomer: δ_H_ 4.25 (1H, d, *J* = 10.2 Hz, H-2), 4.36 (1H, d, *J* = 9.6 Hz, H-3), 4.41 (1H, d, *J* = 8.4 Hz, H-4), 5.88 (1H, d, *J* = 2.4 Hz, H-6), 5.78 (1H, d, *J* = 2.4 Hz, H-8), 6.73 (1H, d, *J* = 2.4 Hz, H-10), 6.67 (1H, d, *J* = 7.8 Hz, H-13), 6.46 (1H, dd, *J* = 8.4, 1.8 Hz, H-14), 4.54 (1H, d, *J* = 7.2 Hz, H-2′), 3.79 (1H, m H-3′), 2.48 (1H, dd, *J* = 16.2, 7.8 Hz, H-4′α), 2.76 (1H, dd, *J* = 16.2, 5.4 Hz, H-4′β), 6.07 (1H, s, H-6′), 6.59 (1H, d, *J* = 2.4 Hz, H-10′), 6.67 (1H, d, *J* = 8.4 Hz, H-13′), 6.25 (1H, dd, *J* = 8.4, 1.8 Hz, H-14′), minor isomer: δ_H_ 4.34 (1H, d, *J* = 7.8 Hz, H-2), 4.51 (1H, d, *J* = 5.4 Hz, H-3), 4.49 (1H, d, *J* = 7.8 Hz, H-4), 5.83 (1H, d, *J* = 2.4 Hz, H-6), 5.81 (1H, d, *J* = 2.4 Hz, H-8), 6.95 (1H, d, *J* = 1.8 Hz, H-10), 6.76 (1H, d, *J* = 7.8 Hz, H-13), 6.83 (1H, dd, *J* = 6.0, 1.8 Hz, H-14), 4.74 (1H, d, *J* = 7.2 Hz, H-2′), 4.07 (1H, m H-3′), 2.58 (1H, dd, *J* = 16.2, 7.8 Hz, H-4′α), 2.82 (1H, dd, *J* = 16.2, 5.4 Hz, H-4′β), 5.94 (1H, s, H-6′), 6.95 (1H, d, *J* = 1.8 Hz, H-10′), 6.76 (1H, d, *J* = 8.4 Hz, H-13′), 6.82 (1H, dd, *J* = 6.0, 2.4 Hz, H-14′), ^13^C-NMR (CD_3_OD, 150 MHz), major isomer: δ_C_ 83.9 (C-2), 73.6 (C-3), 38.5 (C-4), 107.1 (C-4a), 157.1 (C-5), 97.3 (C-6), 157.1 (C-7), 96.8 (C-8), 158.6 (C-8a), 132.6 (C-9), 116.4 (C-10), 145.6 (C-11), 146.0 (C-12), 116.1 (C-13), 120.5 (C-14), 82.4 (C-2′), 68.9 (C-3′), 28.7 (C-4′), 102.2 (C-4′a), 154.8 (C-5′), 96.0 (C-6′), 155.8 (C-7′), 108.1 (C-8′), 155.6 (C-8′a), 131.8 (C-9′), 115.5 (C-10′), 145.4 (C-11′), 145.7 (C-12′), 116.0 (C-13′), 119.8 (C-14′), minor isomer: δ_C_ 84.0 (C-2), 73.6 (C-3), 38.5 (C-4), 107.1 (C-4a), 157.2 (C-5), 97.5 (C-6), 157.4 (C-7), 96.2 (C-8), 158.6 (C-8a), 132.4 (C-9), 116.1 (C-10), 146.1 (C-11), 146.3 (C-12), 116.0 (C-13), 121.0 (C-14), 82.9 (C-2′), 68.5 (C-3′), 28.4 (C-4′), 100.4 (C-4′a), 154.9 (C-5′), 95.5 (C-6′), 155.7 (C-7′), 108.3 (C-8′), 155.6 (C-8′a), 132.1 (C-9′), 115.1 (C-10′), 146.1 (C-11′), 146.3 (C-12′), 115.9 (C-13′), 120.1 (C-14′).

*Rhodonidin A* (**3**). Yellew amorphous powder; ${\left[\alpha \right]}_{D}^{25}$ −13.2° (*c* = 0.1, MeOH); UV (MeOH) λ_max_ (log ε) 216 (3.39), 278 (2.72) nm; CD (MeOH, *c* = 0.01) nm (ε) 216 (−4.75), 248 (−1.75), 275 (+1.12); IR (KBr) cm^−1^: 3444, 1843, 1714, 1643, 1577, 1558, 1541, 1519, 1489, 1456, 1384, 1338, 1284, 1249, 1195, 1114, 1068, 1033, 815, 636, 455, 443; HRESI-MS: [M − H]^–^, 575.1192 *m*/*z*, (calcd for C_30_H_23_O_12_: 575.1195); ^1^H-NMR (CD_3_OD, 600 MHz) δ_H_ 3.97 (1H, d, *J* = 2.4 Hz, H-2), 3.98 (1H, m, H-3), 2.94 (1H, dd, *J* = 14.4 5.4 Hz,H-4β), 2.52 (1H, dd, *J* = 14.4 9.0 Hz, H-4α), 5.90 (1H, d, *J* = 3.2 Hz, H-6), 5.54 (1H, d, *J* = 3.3 Hz, H-8), 2.68 (1H, d, *J* = 11.4 Hz, H-10β), 2.49 (1H, d, *J* = 11.4 Hz, H-10α), 6.43 (1H, s, H-13), 4.92 (1H, d, *J* = 7.2 Hz, H-2′), 4.11 (1H, m, H-3′), 2.86 (1H, dd, *J* = 16.2, 4.8 Hz, H-4′β), 2.60 (1H, dd, *J* = 16.2, 7.8 Hz, H-4′α), 6.13 (1H, s, H-6′), 6.85 (1H, d, *J* = 2.4 Hz, H-10′), 6.79 (1H, d, *J* = 8.4 Hz, H-13′), 6.74 (1H, dd, *J* = 8.4, 2.4 Hz, H-14′); ^13^C-NMR (CD_3_OD, 150 MHz) δ_C_ 79.5 (C-2), 66.8 (C-3), 27.8 (C-4), 100.4 (C4a), 157.6 (d, C-5), 97.0 (C-6), 157.9 (C-7), 95.8 (C-8), 156.3 (C8a), 89.8 (C-9), 45.9 (C-10), 95.3 (C-11), 194.1 (C-12), 112.8 (C-13), 164.4 (C-14), 83.4 (C-2′), 67.8 (C-3′), 28.3 (C-4′), 103.9 (C-4′a), 166.2 (C-5′), 90.9 (C-6′), 168.0 (C-7′), 105.6 (C-8′), 155.1 (C-8′a), 131.2 (C-9′), 114.8 (C-10′), 146.4 (C-11′), 146.5 (C-12′), 116.3 (C-13′), 119.7 (C-14′).

*Epicatechin-(4*β*→8)-catechin-(4*α*→8)-catechin* (Procyanidin C4) (**4**). White amorphous powder; ${\left[\alpha \right]}_{D}^{25}$ −15.7° (*c* = 0.1, MeOH); UV (MeOH) λ_max_ (log ε) 230 (3.58), 280 (3.31) nm; CD (MeOH, *c* = 0.017) nm (ε) 216 (+6.69), 232 (+9.49), 274 (−0.35), 312 (−0.35); ESI-MS: [M]^–^, 866.2 *m*/*z*, [M + H + Na]^+^ 890.1 *m*/*z* (calcd for C_45_H_3__8_O_18_: 866.2); ^1^H-NMR (CD_3_OD, 600 MHz) δ_H_ 5.24 (1H, s, H-2), 3.98 (1H, brs, H-3), 4.75 (1H, d, *J* = 1.2 Hz, H-4), 5.84 (2H, s, H-6, H-8), 7.00 (1H, d, *J* = 1.8 Hz, H-10), 6.74 (1H, d, *J* = 8.4 Hz, H-13), 6.67 (1H, dd, *J* = 8.4, 1.8 Hz, H-14), 4.48 (1H, d, *J* = 10.2 Hz, H-2′), 4.68 (1H, m, H-3′), 4.71 (1H, d, *J* = 7.2 Hz, H-4′), 5.90 (1H, s, H-6′), 7.01 (1H, d, *J* = 1.8 Hz, H-10′), 6.81 (1H, d, *J* = 8.4 Hz, H-13′), 6.88 (1H, dd, *J* = 6.0, 1.8 Hz, H-14′), 4.14 (1H, m, H-3′′), 2.68 (1H, dd, *J* = 16.8, 5.4 Hz, H-4′′α), 2.62 (1H, dd, *J* = 16.8, 6.0 Hz, H-4′′β), 5.97 (1H, s, H-6′′), 6.90 (1H, brs, H-10′′), 6.71 (1H, d, *J* = 7.2 Hz, H-13′′), 6.88 (1H, d, *J* = 6.0, 1.8 Hz, H-14′′); ^13^C-NMR (CD_3_OD, 150 MHz) δ_C_ 77.3 (C-2), 72.7 (C-3), 37.0 (C-4), 101.2 (C-4a), 157.3 (C-5), 97.7 (C-6), 158.4 (C-7), 96.1 (C-8), 157.1 (C-8a), 132.9 (C-9), 115.0 (C-10), 145.2 (C-11), 145.8 (C-12), 116.1 (C-13), 118.7 (C-14), 83.5 (C-2′), 73.0 (C-3′), 39.0 (C-4′), 107.2 (C-4′a), 156.4 (C-5′), 97.2 (C-6′), 156.0 (C-7′), 107.7 (C-8′), 156.9 (C-8′a), 132.1 (C-9′), 116.4 (C-10′), 146.3 (C-11′), 146.1 (C-12′), 115.9 (C-13′), 121.3 (C-14′), 82.0 (C-2′′), 68.3 (C-3′′), 26.8 (C-4′′), 100.6 (C-4′′a), 155.7 (C-5′′), 99.4 (C-6′′), 155.6 (C-7′′), 107.3 (C-8′′), 155.0 (C-8′′a), 132.6 (C-9′′), 114.5 (C-10′′), 145.8 (C-11′′), 146.5 (C-12′′), 116.1 (C-13′′), 119.4 (C-14′′).

*Epicatechin-(4*β*→8;2*β*→O→7)-epicatechin-(4*β*→8)-catechin* (Cinnamtannin D1) (**5**). White amorphous powder; ${\left[\alpha \right]}_{D}^{25}$ +34.5° (*c* = 0.1, MeOH); UV (MeOH) λ_max_ (log ε) 230 (3.56), 243 (3.57), 280 (3.45) nm; CD (MeOH, *c* = 0.01) nm (ε) 222 (−5.17), 241 (+13.35), 272, (−1.8), 304 (+0.49); IR (KBr) cm^−1^: 3383, 1614, 1558, 1521, 1508, 1448, 1373, 1338, 1284, 1246, 1211, 1178, 1143, 1114, 1064, 1010, 974, 869, 819, 781, 451, 418; ESI-MS: [M − H]^–^, 863.1 *m*/*z*, [M + Na]^+^ 887.1 *m*/*z* (calcd for C_45_H_36_O_18_: 864.1); ^1^H-NMR (CD_3_OD, 600 MHz) δ_H_ 3.46 (1H, d, *J* = 3.6 Hz, H-3), 4.00 (1H, d, *J* = 3.6 Hz, H-4), 5.94 (1H, s, H-6), 6.01 (1H, s, H-8), 7.09 (1H, d, *J* = 1.8 Hz, H-10), 6.85 (1H, d, *J* = 8.4 Hz, H-13), 6.95 (1H, dd, *J* = 8.4, 2.4 Hz, H-14), 5.51 (1H, brs, H-2′), 4.06 (1H, d, *J* = 1.8 Hz, H-3′), 4.53 (1H, brs, H-4′), 5.84 (1H, s, H-6′), 7.23 (1H, d, *J* = 1.8 Hz, H-10′), 6.84 (1H, d, *J* = 8.4 Hz, H-13′), 7.09 (1H, dd, *J* = 6.0, 1.8 Hz, H-14′), 3.95 (1H, d, *J* = 9.0 Hz, H-2′′), 3.67 (1H, m, H-3′′), 3.05 (1H, dd, *J* = 16.2, 6.0 Hz, H-4′′α), 2.42 (1H, dd, *J* = 16.2, 10.2 Hz, H-4′′β), 6.10 (1H, s, H-6′′), 6.75 (1H, d, *J* = 2.4 Hz, H-10′′), 6.75 (1H, d, *J* = 8.4 Hz, H-13′′), 6.67 (1H, d, *J* = 7.8, 1.8 Hz, H-14′′); ^13^C-NMR (CD_3_OD, 150 MHz) δ_C_ 100.0 (C-2), 67.1 (C-3), 28.8 (C-4), 104.9 (C-4a), 156.5 (C-5), 98.3 (C-6), 157.6 (C-7), 96.3 (C-8), 154.1 (C-8a), 132.4 (C-9), 115.7 (C-10), 145.4 (C-11), 146.6 (C-12), 116.2 (C-13), 120.0 (C-14), 78.6 (C-2′), 72.4 (C-3′), 38.2 (C-4′), 106.5 (C-4′a), 155.7 (C-5′), 95.8 (C-6′), 150.9 (C-7′), 106.2 (C-8′), 151.7 (C-8′a), 131.5 (C-9′), 116.5 (C-10′), 145.8 (C-11′), 146.2 (C-12′), 116.0 (C-13′), 121.0 (C-14′), 83.2 (C-2′′), 70.0 (C-3′′), 30.6 (C-4′′), 101.7 (C-4′′a), 155.3 (C-5′′), 96.3 (C-6′′), 155.5 (C-7′′), 108.7 (C-8′′), 155.3 (C-8′′a), 132.6 (C-9′′), 116.0 (C-10′′), 145.7 (C-11′′), 145.9 (C-12′′), 115.8 (C-13′′), 119.9 (C-14′′).

### 3.4. Total Antioxidant Capacity (TAC)

Pure compounds were tested by using the CUPric Reducing Antioxidant Capacity (CUPRAC) method [28] according to the protocol of QuantiChrom Antioxidant Assay kit (Bioassay Systems, Hayward, CA, USA) [29]. These assays are based on the reduction of Cu^2+^ to Cu^+^ by the combined action of all antioxidants (reducing agents) in a sample. The resulting Cu^+^ specifically forms a colored complex with a dye agent (4,4′-dicarboxy-2,2′-biquinoline) and the color intensity at 570 nm is measured as TAC. Briefly, compounds were diluted with distilled water to produce solutions of 0.1, 0.25, 0.5, and 1 mM concentration. The reaction was initiated by the addition of 100 µL mixture of copper sulfate and dye agent with 20 µL of each compound solution. The absorbance at 570 nm was calculated for each concentration relative to a blank absorbance and was plotted as a function of concentration of standard Trolox. At least three independent determinations were performed. The antioxidant activities of purified compounds **1**–**5** are expressed as TEAC (Trolox Equivalent Antioxidant Activity) values in comparison with TEAC activity of reported reference compounds, catechin (Sigma-Aldrich, USA) and epicatechin (Sigma-Aldrich, USA). Trolox was employed at concentrations ranging from 10–1000 μM to construct a calibration curve. TEAC value is defined as the concentration of standard Trolox solution with equivalent activity to 1 mM concentration solution of purified compound.

### 3.5. Free Radical Scavenging Activity

The free radical scavenging activities of purified compounds were determined according to previous report. Briefly, the reaction for scavenging DPPH radicals was carried out by adding 2 μL sample to 198 μL DPPH solution (100 μM) at 25 °C. The mixture was shaken vigorously and left to stand for 30 min in the dark before measuring the absorbance at 517 nm against a blank. For the radical scavenging activities of procyanidins, EC_50_ values were calculated as the concentrations (μM) that inhibited 50% of the DPPH radicals in the reaction.

Scavenging ability (%) = [(ΔA_517_ of control − ΔA_517_ of sample)/ΔA_517_ of control] × 100

For comparison purposes, standard antioxidant compound trolox (from 0.7815–100 μM, R^2^ = 0.999) was used as standard antioxidant compound.

### 3.6. Antibacterial Activity

Eight strains of microorganisms were used: *Bacillus cereus* (ATCC 9139), *Enterococcus faecalis* (ATCC 29212), *Escherichia coli* (ATCC 35150), *Listeria monocytogenes* (ATCC 7644), *Pseudomonas aeruginosa* (ATCC 27853), *Salmonella enterica* (ATCC 13311), *Staphylococcus aureus* (ATCC 43300), and *Helicobacter pylori* (ATCC 700392), which were employed to evaluate the antibacterial assay. Minimum inhibitory concentration (MIC) and minimum bactericidal concentration (MBC) were determined by the broth micro-dilution method according to the guidelines of the Clinical and Laboratory Standards Institute [30]. *H. pylori* was grown on blood agars under microaerophilic conditions at 37 °C for 48–72 h while other bacteria strains were cultured on nutrient agar (Difco, USA) and incubated at 37 °C for 24 h. Bacterial inoculums were prepared in normal saline and diluted to give a final density of 5 × 10^5^ cfu/mL. All compounds were dissolved in DMSO (Sigma, USA) and then in nutrient broth to reach a final concentration of 512 µg/mL. Serial two-fold dilutions were made in a concentration range from 0.25–256 µg/mL. The MIC and MBC were defined as the lowest concentration at which no visible growth occurred in comparison with antibiotics (ampicillin, tetracyclin and metronidazole) as a positive control. Tests were repeated three times for each compound.

## 4. Conclusions

Five compounds, including two procyanidin dimers, procyanidin A1 (**1**) and B3 (**2**), two procyanidin trimmers, procyanidin C4 (**4**) and cinnamtannin D1 (**5**), and one new procyanidin dimer, rhodonidin A (**3**), have been isolated from the leaves of *R. formosanum*. Compound **3** demonstrated strong antimicrobial activity against *Staphylococcus aureus* at MIC value of 4 μg/mL. Compounds **1**–**5** also showed pronounced antioxidant activities. The pleiotropic effects of procyanidins isolated from the leaves of *R. formosanum* can be a source of promising compounds for the development of future pharmacological applications.

## Supplementary Materials

Supplementary materials can be accessed at: http://www.mdpi.com/1420-3049/20/07/12787/s1.

## References

[1] Maatta-Riihinen K.R., Kahkonen M.P., Torronen A.R., Heinonen I.M. (2005). Catechins and procyanidins in berries of *Vaccinium* species and their antioxidant activity. J. Agric. Food Chem..

[2] Fu C.L., Wang H.Y., Ng W.L., Song L.X., Huang D.J. (2013). Antioxidant activity and proanthocyanidin profile of *Selliguea feei* rhizomes. Molecules.

[3] He F., Pan Q.H., Shi Y., Duan C.Q. (2008). Biosynthesis and genetic regulation of proanthocyanidins in plants. Molecules.

[4] Zang X.Y., Shang M.Y., Xu F., Liang J., Wang X., Mikage M., Cai S.Q. (2013). A-type proanthocyanidins from the stems of *Ephedra sinica* (Ephedraceae) and their antimicrobial activities. Molecules.

[5] Kresty L.A., Howell A.B., Baird M. (2011). Cranberry proanthocyanidins mediate growth arrest of lung cancer cells through modulation of gene expression and rapid induction of apoptosis. Molecules.

[6] Wang H.Y., Song L.X., Feng S.B., Liu Y.C., Zuo G., Lai F.L., He G.Y., Chen M.J., Huang D.J. (2013). Characterization of proanthocyanidins in stems of *Polygonum multiflorum* thunb as strong starch hydrolase inhibitors. Molecules.

[7] Chou S.C., Krishna V., Chou C.H. (2009). Hydrophobic Metabolites from *Rhododendron formosanum* and their Allelopathic Activities. Nat. Prod. Commun..

[8] Krishna V., Chang C.I., Chou C.H. (2006). Two isomeric epoxysitosterols from Rhododendron formosanum: ^1^H- and ^13^C-NMR chemical shift assignments. Magn. Reson. Chem..

[9] Way T.D., Tsai S.J., Wang C.M., Ho C.T., Chou C.H. (2014). Chemical constituents of *Rhododendron formosanum* show pronounced growth inhibitory effect on non-small-cell lung carcinoma cells. J. Agric. Food Chem..

[10] Chou S.C., Huang C.H., Hsu T.W., Wu C.C., Chou C.H. (2010). Allelopathic potential of *Rhododendron formosanum* Hemsl in Taiwan. Allelopathy J..

[11] Wang C.M., Li T.C., Jhan Y.L., Weng J.H., Chou C.H. (2013). The impact of microbial biotransformation of catechin in enhancing the allelopathic effects of *Rhododendron formosanum*. PLoS ONE.

[12] Botha J.J., Ferreira D., Roux D.G. (1978). Condensed tannins-circular-dichroism method of assessing absolute-configuration at C-4 of 4-arylflavan-3-ols, and stereochemistry of their formation from flavan-3,4-diols. J. Chem. Soc. Chem. Comm..

[13] Barrett M.W., Klyne W., Scopes P.M., Fletcher A.C., Porter L.J., Haslam E. (1979). Plant proanthocyanidins. Part 6. chiroptical studies. Part 95. Circular-Dichroism of procyanidins. J. Chem. Soc. Perkin Trans. 1.

[14] Lou H.X., Yamazaki Y., Sasaki T., Uchida M., Tanaka H., Oka S. (1999). A-type proanthocyanidins from peanut skins. Phytochemistry.

[15] Oizumi Y., Mohri Y., Hirota M., Makabe H. (2010). Synthesis of procyanidin B3 and its anti-inflammatory activity. The effect of 4-alkoxy group of catechin electrophile in the Yb(OTf)(3)-catalyzed condensation with catechin nucleophile. J. Org. Chem..

[16] Guyot S., Vercauteren J., Cheynier V. (1996). Structural determination of colourless and yellow dimers resulting from (+)-catechin coupling catalysed by grape polyphenoloxidase. Phytochemistry.

[17] Yan G.Q., Li S.P., Hu J., Zhai X.Y., Ma W., Li N., Wang K.J. (2014). Phenolic constituents from the roots of *Rosa laevigata* (Rosaceae). Biochem. Syst. Ecol..

[18] Karioti A., Bilia A.R., Gabbiani C., Messori L., Skaltsa H. (2009). Proanthocyanidin glycosides from the leaves of *Quercus ilex* L. (Fagaceae). Tetrahedron Lett..

[19] Slade D., Ferreira D., Marais J.P.J. (2005). Circular dichroism, a powerful tool for the assessment of absolute configuration of flavonoids. Phytochemistry.

[20] Saito A., Doi Y., Tanaka A., Matsuura N., Ubukata M., Nakajima N. (2004). Systematic synthesis of four epicatechin series procyanidin trimers and their inhibitory activity on the Maillard reaction and antioxidant activity. Bioorg. Med. Chem..

[21] Lin H.C., Lee S.S. (2010). Proanthocyanidins from the leaves of *Machilus philippinensis*. J. Nat. Prod..

[22] Killday K.B., Davey M.H., Glinski J.A., Duan P.G., Veluri R., Proni G., Daugherty F.J., Tempesta M.S. (2011). Bioactive A-type proanthocyanidins from *Cinnamomum cassia*. J. Nat. Prod..

[23] Kamiya K., Ohno A., Horii Y., Endang H., Umar M., Satake T. (2003). A-type proanthocyanidins from the bark of *Parameria laevigata*. Heterocycles.

[24] De Bruyne T., Pieters L., Witvrouw M., de Clercq E., vanden Berghe D., Vlietinck A.J. (1999). Biological evaluation of proanthocyanidin dimers and related polyphenols. J. Nat. Prod..

[25] Ming D.S., Lopez A., Hillhouse B.J., French C.J., Hudson J.B., Towers G.H.N. (2002). Bioactive constituents from *Iryanthera megistophylla*. J. Nat. Prod..

[26] Ricardo-Da-Silva J.M., Darmon N., Fernandez Y., Mitjavila S. (1991). Oxygen free-radical scavenger capacity in aqueous models of different procyanidins from grape seeds. J. Agric. Food Chem..

[27] Wang C.M., Chen H.T., Li T.C., Weng J.H., Jhan Y.L., Lin S.X., Chou C.H. (2014). The role of pentacyclic triterpenoids in the allelopathic effects of *Alstonia scholaris*. J. Chem. Ecol..

[28] Ozyurek M., Guclu K., Apak R. (2011). The main and modified CUPRAC methods of antioxidant measurement. TrAC Trend. Anal. Chem..

[29] Prior R.L., Wu X.L., Schaich K. (2005). Standardized methods for the determination of antioxidant capacity and phenolics in foods and dietary supplements. J. Agric. Food Chem..

[30] CLSI (2015). Methods for Dilution Antimicrobial Susceptibility Tests for Bacteria that Grow Aerobically.

