# Supplementary material for: Structure Elucidation of Procyanidins Isolated from Rhododendron formosanum and Their Anti-Oxidative and Anti-Bacterial Activities
Source: Molecules. 2015 Jul 15;20(7):12787–803. doi: 10.3390/molecules200712787 (PMC6332352; doi:10.3390/molecules200712787)
Supplement: Supplementary file 1 [file molecules-20-12787-s001.pdf]

Supporting Materials

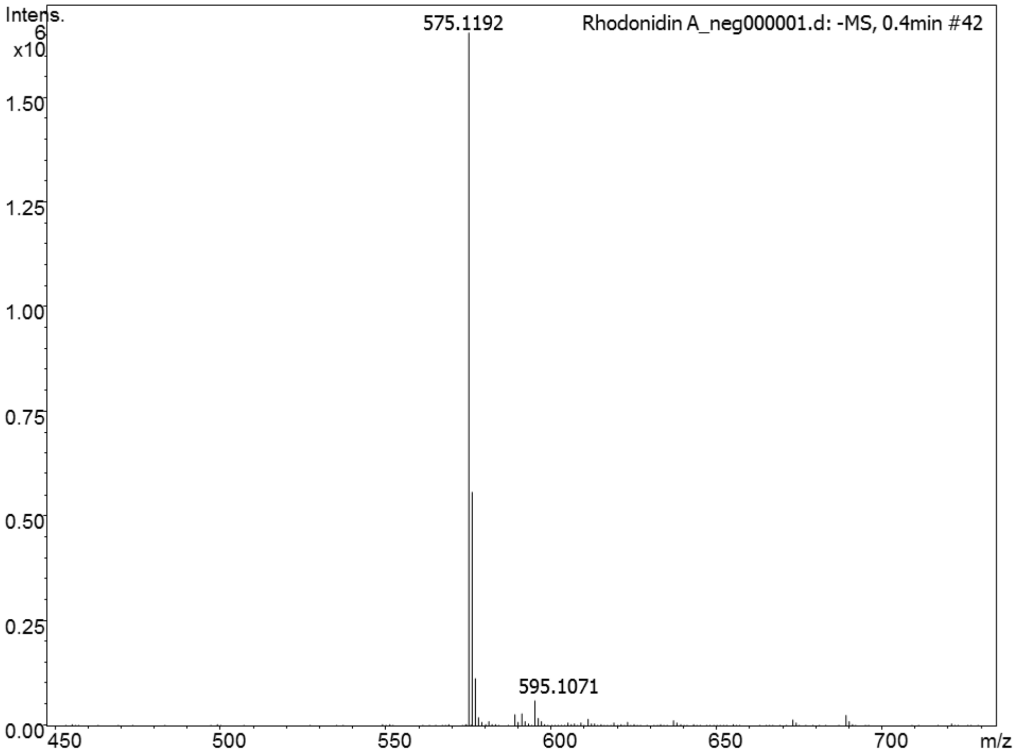

Figure S1. HR-ESIMS of **3**

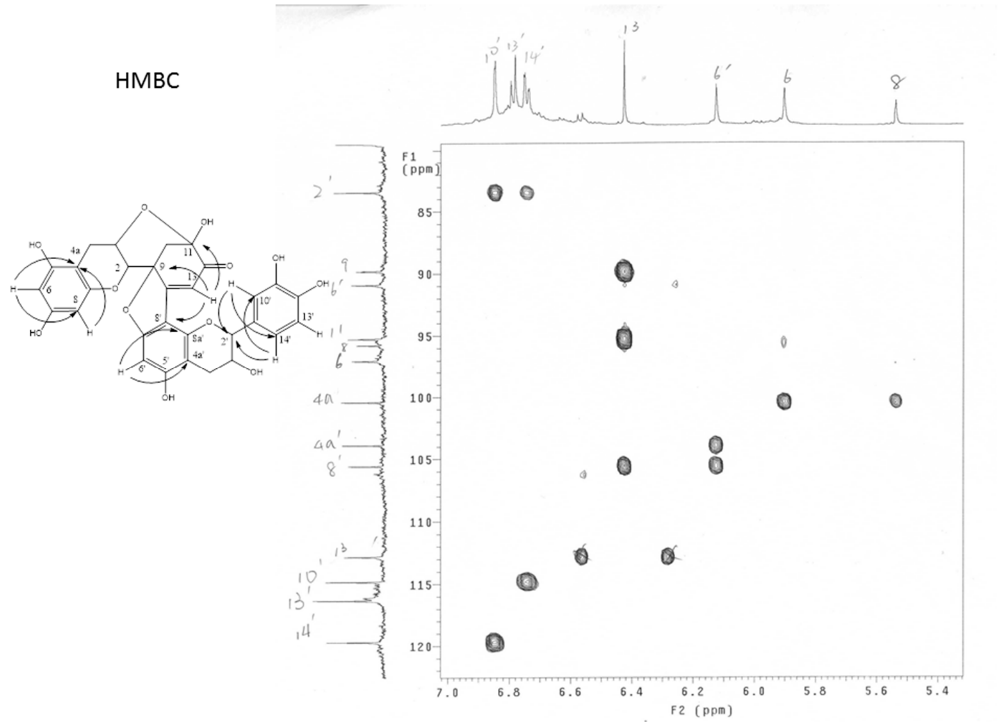

Figure S2. HMBC correlation of **3**.

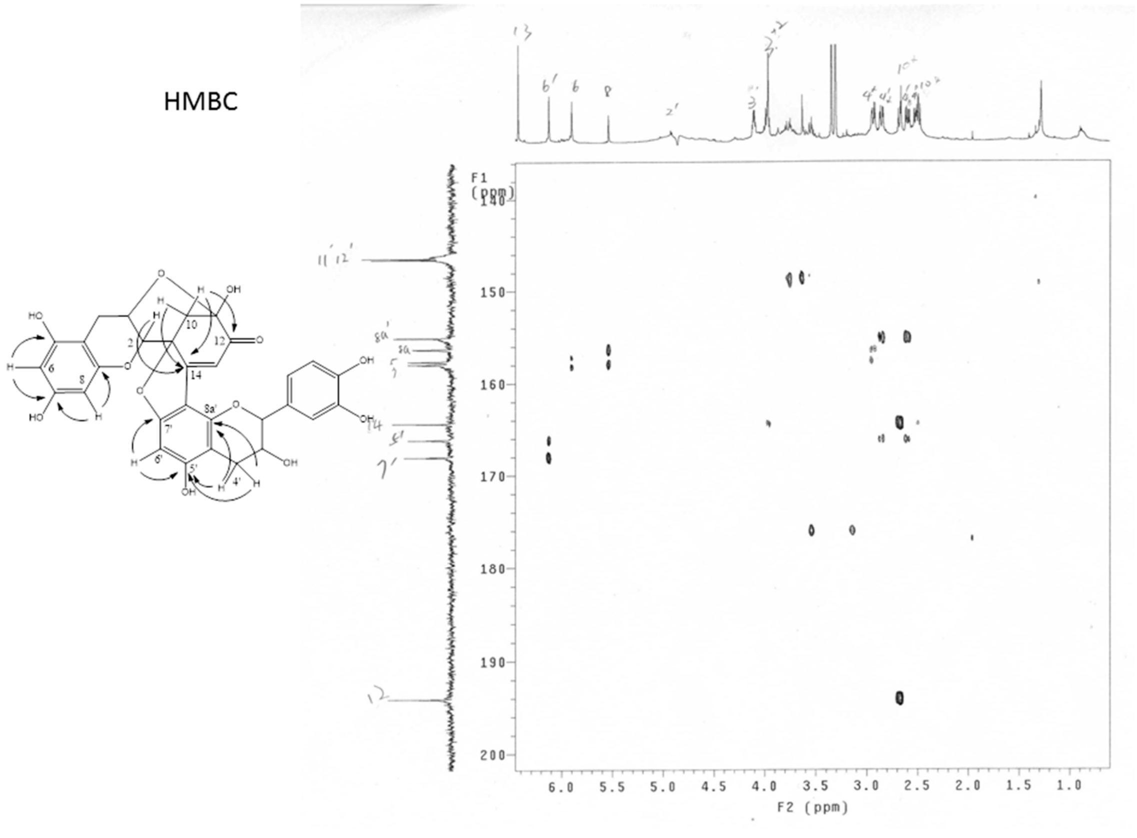

Figure S3. HMBC correlation of **3**, continued.

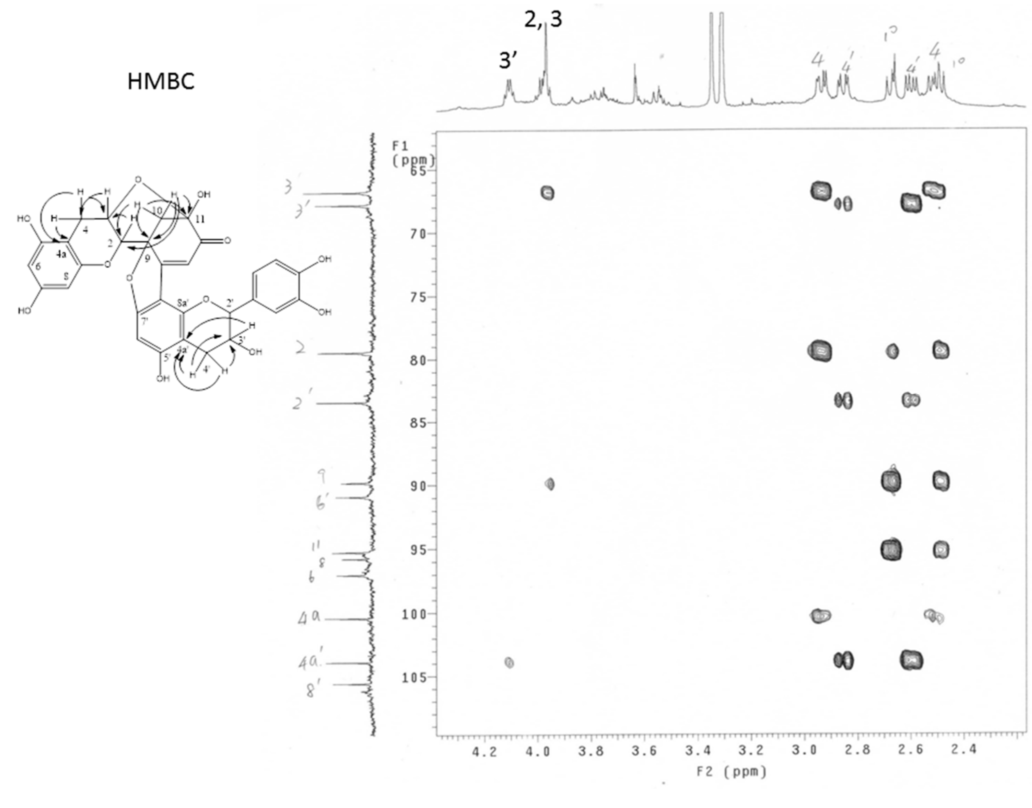

Figure S4. HMBC correlation of **3**, continued.

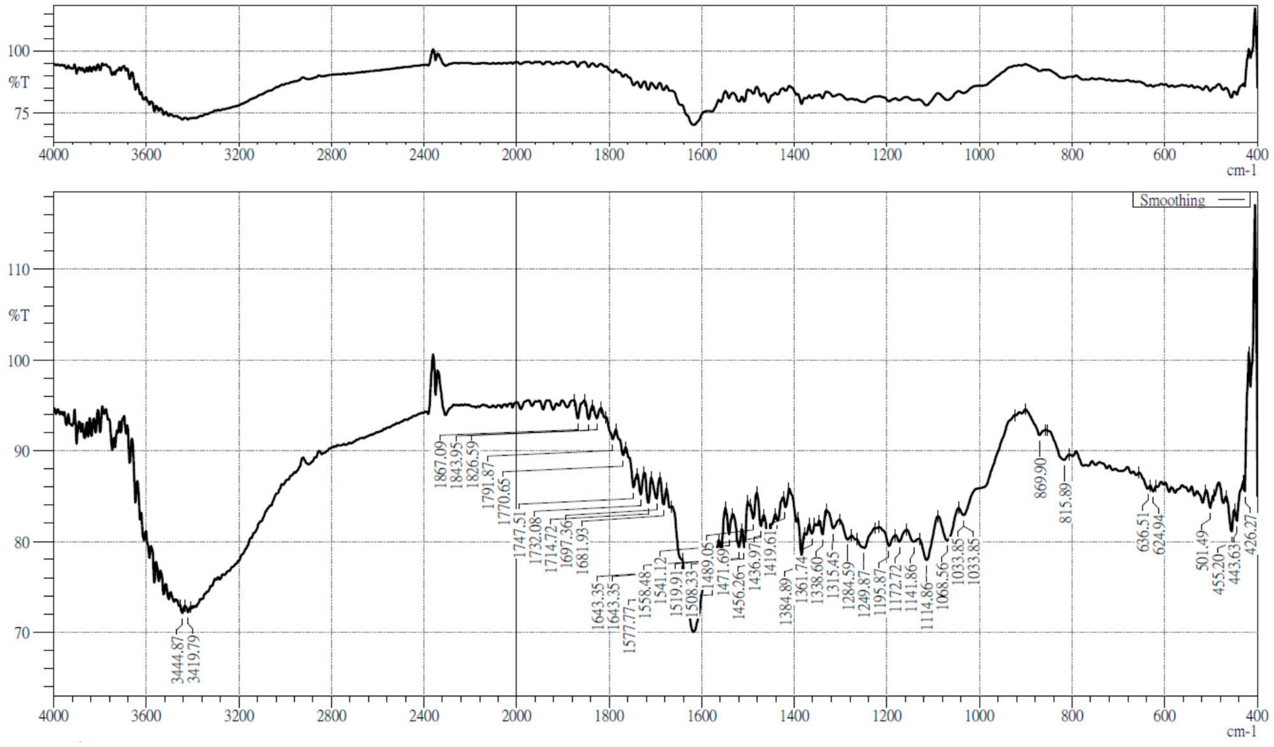

Figure S5. FT-IR spectrum of **3**.

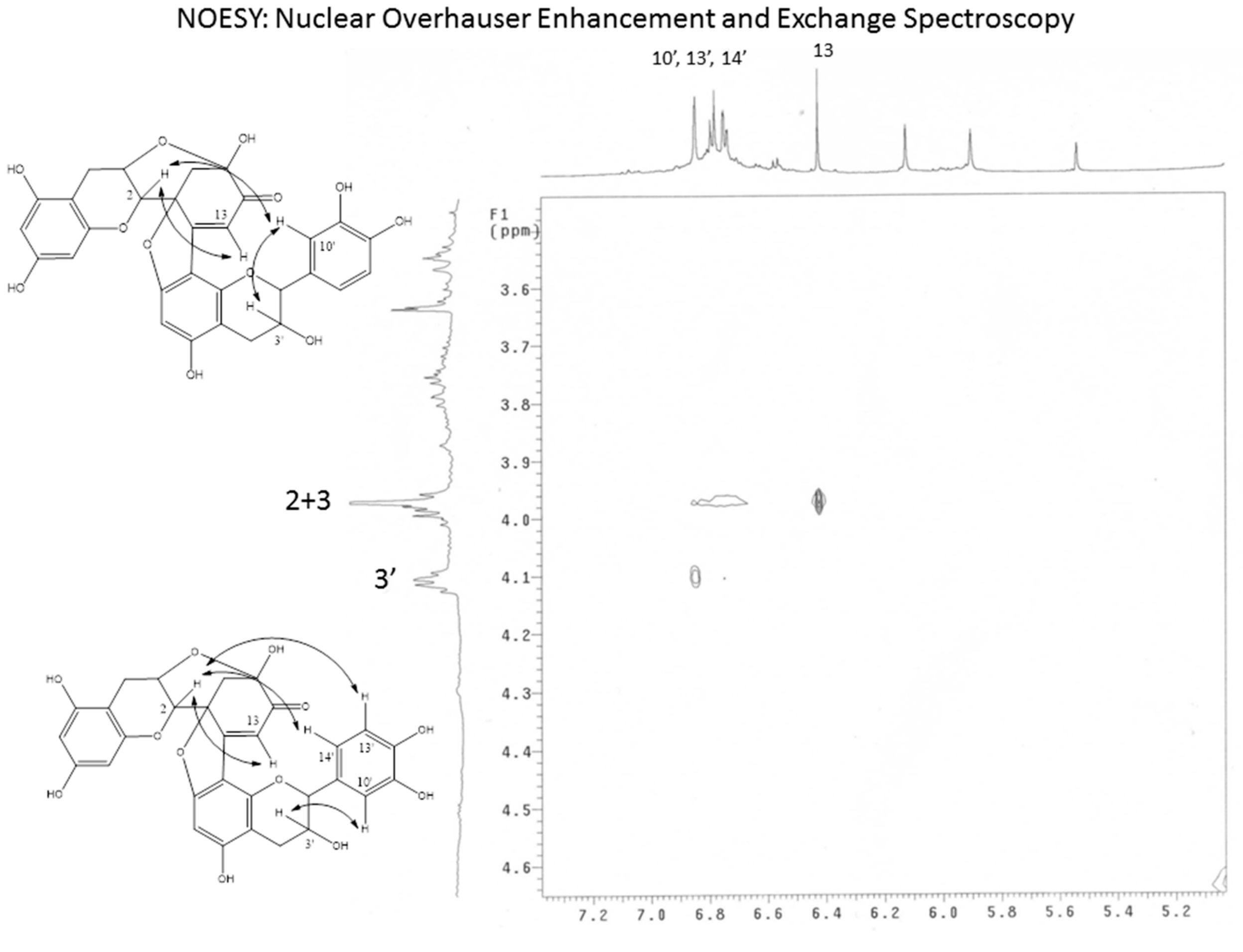

Figure S6. Selected NOE correlations of **3**.
